# Preclinical Models and Promising Pharmacotherapeutic Strategies in Liver Fibrosis: An Update

**DOI:** 10.3390/cimb45050270

**Published:** 2023-05-11

**Authors:** Tea Omanovic Kolaric, Lucija Kuna, Marina Covic, Hrvoje Roguljic, Anita Matic, Renata Sikora, Marija Hefer, Ana Petrovic, Vjera Mihaljevic, Robert Smolic, Ines Bilic-Curcic, Aleksandar Vcev, Martina Smolic

**Affiliations:** 1 Department of Pharmacology and Biochemistry, Faculty of Dental Medicine and Health, 31000 Osijek, Croatia; tomanovic@fdmz.hr (T.O.K.); lkuna@fdmz.hr (L.K.); marinacovic19@gmail.com (M.C.); hroguljic@mefos.hr (H.R.); ana.petrovic@fdmz.hr (A.P.); vnincevic@fdmz.hr (V.M.); rsmolic@fdmz.hr (R.S.); 2 Department of Pharmacology, Faculty of Medicine, 31000 Osijek, Croatia; anita.matic@fdmz.hr (A.M.); ibcurcic@mefos.hr (I.B.-C.); 3 Department of Internal Medicine, University Hospital Osijek, 31000 Osijek, Croatia; 4 Department of Pathophysiology and Physiology with Immunology, Faculty of Dental Medicine and Health, 31000 Osijek, Croatia; avcev@mefos.hr; 5 Department of Dental Medicine, Faculty of Dental Medicine and Health, 31000 Osijek, Croatia; renata.sikora@fdmz.hr; 6 Department of Physics, Biophysics, and Chemistry, Faculty of Dental Medicine and Health, 31000 Osijek, Croatia; marija.hefer@fdmz.hr; 7 Department of Endocrinology, University Hospital Osijek, 31000 Osijek, Croatia

**Keywords:** liver fibrosis, hepatic stellate cells, NAFLD

## Abstract

Liver fibrosis represents one of the greatest challenges in medicine. The fact that it develops with the progression of numerous diseases with high prevalence (NAFLD, viral hepatitis, etc.) makes liver fibrosis an even greater global health problem. Accordingly, it has received much attention from numerous researchers who have developed various in vitro and in vivo models to better understand the mechanisms underlying fibrosis development. All these efforts led to the discovery of numerous agents with antifibrotic properties, with hepatic stellate cells and the extracellular matrix at the center of these pharmacotherapeutic strategies. This review focuses on the current data on numerous in vivo and in vitro models of liver fibrosis and on various pharmacotherapeutic targets in the treatment of liver fibrosis.

## 1. Introduction

Liver fibrosis is one of the emerging global health issues. Many factors contribute to this, such as an increase in incidence and prevalence of underlying conditions (non-alcoholic fatty liver disease—NAFLD, chronic viral hepatitis, exposure to toxins, polypharmacy, etc.), lack of adequate diagnostic tools for aforementioned diseases, unclear mechanisms underlying hepatic fibrosis, and lack of clinically approved and effective pharmacotherapeutic options [1,2,3,4,5]. As a common outcome of various chronic liver diseases, it can also, if left uncontrolled, eventually progress to cirrhosis, and finally increase the risk for the development of hepatocellular carcinoma [4,6]. The last two diseases are the major causes of death from chronic liver diseases [4]. Therefore, there is a clear necessity to better understand, diagnose, and treat fibrosis.

NAFLD, one of the underlying conditions for the development of fibrosis, is considered the most common form of chronic liver disease in Western countries. In fact, NAFLD affects 20–30% of adults worldwide, and its prevalence is expected to increase in the coming decades [6,7]. However, no adequate pharmacotherapeutic solution has been approved for the treatment of this disease. There is also a deficiency in its diagnostics, with no specific markers being introduced to common clinical practice. Additionally, according to Williams et al., 73% of patients presenting with cirrhosis or liver failure on their first admission were never referred to a liver clinic, suggesting that liver disease is not detected early enough [8].

The basic pathophysiological process in the development of liver fibrosis involves the excessive accumulation of the extracellular matrix (ECM) and its components (extracellular proteins, proteoglycans, and carbohydrates) in the liver, which is responsible for liver fibrosis development [9,10]. Numerous cells are involved in this process, but the exact molecular mechanisms have not yet been fully elucidated. From this issue arises the need to develop more reliable in vivo and in vitro models that could serve not only to better understand fibrosis development itself but also to discover new pharmacotherapeutic targets. According to one study, 73% of patients are diagnosed with liver disease at later stages, and etiology-specific interventions are often too slow to prevent serious complications and cannot completely reverse fibrosis [6,8,11,12]. Therefore, antifibrotic drugs are the focus of numerous researchers.

This review presents current data on in vivo and in vitro models of liver fibrosis and pharmacotherapeutic targets in the treatment of liver fibrosis.

## 2. Preclinical Models of Liver Fibrosis

### 2.1. In Vitro Models of Liver Fibrosis

One of the first described in vitro liver fibrosis models was on the primary rodent hepatic stellate cells (HSCs) such as the murine cell line (GRX) originating from mice infected with *Schistosoma mansoni*, cirrhotic fat-storing cells (CFSCs), and normal fat-storing (NFSC) derived from healthy and cirrhotic livers from Wistar rats [13,14]. On the other hand, the first human HSC line was LI90. This cell line was derived from an epithelioid hemangioendothelioma. Although these cells represented a good model for the observation of drug targets in HSCs activation, LI90 cells undergo a deterioration process after a few passages [13].

Primary HSCs originating from normal liver tissue are used as a model for the screening of pro- and antifibrosis compounds and represent a significant reflection of in vivo liver fibrosis [13,15]. Moreover, HSCs are the cell type leading to scar formation in almost all types of liver injuries [16,17]. Liver fibrosis involves HSC activation from quiescent HSCs responsible for vitamin A storage to a profibrogenic myofibroblast phenotype [13]. Additionally, all myofibroblasts are identified based on proteins that represent their activation phenotype of scar tissue formation such as osteopontin, collagen types I and III, lysyl oxidase TIMP1 (tissue inhibitor of metalloproteinase 1), and αSMA (alpha-smooth muscle actin) [18]. In fact, these markers have a key role in the activation of HSCs and the determination of the formation of fibrosis in cell culture or liver tissues [17].

Although HSC activation can be obtained in culture, recent studies have shown that the mechanisms by which it is mediated can be significantly different. In fact, the life span of HSCs as primary cells is limited, which interferes with their use. Regardless of increased purity and improvement in isolation techniques, HSC cultures are very easily contaminated with other types of liver cells [13,19]. The purest HSCs are obtained using fluorescence-activated cell sorting (FACS) of non-parenchymal cells (NPCs) such as liver sinusoidal endothelial cells (LSECs), Kupffer cells, and stellate cells [17,20].

HSC activation induced in tissue culture may be avoided by seeding HSCs as 3D spheroids. Treatment with profibrogenic growth factors such as transforming growth factor β-1 (TGFβ-1) or the transfer of formed spheroids in 2D culture represent more controlled activation in 3D cultures whereby the cells remain in a quiescent state [17,21]. However, the establishment of a 2D culture of primary HSCs is easy to obtain, while the establishment of 3D cultures requires a lot of optimization for establishment and analysis. The most significant differences in HSC activation among in vivo and in vitro studies are related to the complex of 3D cell organization and ECM, interaction with other cell types, and the dependence on hepatocyte damage during this process [21]. An improved way to mimic fibrosis in vitro would be a system that involves hepatocytes that, upon injury, cause HSC activation. Furthermore, the co-cultivation of HSCs and hepatocytes was anticipated to improve hepatocyte cultures, as primary hepatocytes rapidly lose their metabolic capacity when placed in 2D [17]. Considering that liver toxicity in humans presents a low correlation with in vivo studies, the use of primary human hepatocytes and NPC is preferred in in vitro toxicity studies and drug-induced liver fibrosis in humans [17,22].

Spheroid co-cultures are formed using HSCs and primary rat hepatocytes via self-forming aggregation techniques. At the same time, in many studies, 3D liver spheroids are introduced using different techniques that involve micromolds made of non-adhesive materials [17,23].

In conclusion, great progress has been achieved in terms of in vitro liver cultures, particularly 3D liver cultures, while the main task is related to the preservation of functional hepatocytes for extended periods. On the other hand, the incorporation of NPCs enables the use of these cultures in the research of liver fibrosis [17,21]. However, further research is needed to elucidate whether systems such as spheroids and bioprinted liver tissue can replace monolayer primary HSC cultures as the most commonly used models of liver fibrosis and help in the advancement of effective strategies for the clinical treatment of liver fibrosis.

The most commonly used cell types for the establishment of the liver fibrosis model, along with their main characteristics, are summarized in Table 1.

### 2.2. In Vivo Models of Liver Fibrosis

In previous in vivo models, several methods of inducing liver fibrosis have been shown to be successful. In some methods, certain shortcomings have been pointed out, and their application depends on the parameters to be observed. Through research on animals, it has been established that sometimes one causative agent is not enough to cause damage to the liver, but it is necessary to combine them in order to achieve a high-quality model of fibrosis [27,28]. The negative side of such a combined model is the fact that certain substances can directly cause other harmful effects on the organism and thus interfere with the analysis itself [29].

One of the most commonly used models of chemical fibrosis and hepatotoxicity in rodents is using carbon tetrachloride (CCL_4_). This model shows a high similarity to human fibrosis, and as such, has become a well-accepted in vivo model [30]. Previous research has been conducted on both rat and mouse models, but mice, especially BALB/c mice, have proven to be a better model due to their higher metabolic rate of CCl_4_ compared to rats [31,32]. The main reason for the success of the CCl_4_ model is the fact that, in the presence of the CYP2E1 enzyme, it is transformed into highly toxic forms of free radicals, trichlormethyl radical (CCl_3_) and trichlormethyl peroxide (CCl_3_O_2_), which results in an increased formation of free radicals and the activation of the lipid peroxidation process. They then participate in the development of the acute phase of the disease, which is characterized by necrosis, the activation of Kupffer cells, and inflammatory response [33]. Another chemical which proved to be a good model, and which, compared to the CCl_4_ model, showed a greater inflammatory response and more progressive ductal hyperplasia, is thioacetamide (TAA). It is a chemical that is not toxic to the liver, but just like the CCl_4_ model, it is catalyzed by CYP450 isoenzymes, and its metabolites bind to proteins and lipids, causing increased oxidative stress and resulting in chronic liver necrosis and fibrosis [34]. Studies comparing the effectiveness of CCl_4_ and TTA have also been conducted. In the primate model Macaca fascicularis, both chemicals showed a high Child–Pugh Score for cirrhosis mortality, but TTA proved to be a more efficient model in creating a more chronic form of liver fibrosis [35].

Considering the confirmed effectiveness of causing liver fibrosis, the use of the carcinogenic chemicals dimethylnitrosamine and diethylnitrosamine for this purpose has increasingly been introduced in various studies. The use of diethylnitrosamine in the development of tumors was shown to be effective in mouse models C3H and B6C3F1. In rat models, R16 proved to be the one that reacted the most to the application of both carcinogenic chemicals [36,37]. In the Spraque–Dawley rat model, it was shown that iron deposition and fat accumulation can potentially be the cause of pathological changes in liver fibrosis [38,39].

Given the fact that one of the most common causes of liver fibrosis is excessive and long-term alcohol consumption, known as alcoholic liver disease, ethanol is one of the potential in vivo models used to induce liver fibrosis in animals [40]. The use of ethanol in the liver causes a decrease in the level of antioxidant enzymes and an increase in radicals that lead to the cell death of hepatocytes, the inflammatory process, and the activation of HSCs and fatty liver [41,42]. The disadvantage of this method is the fact that rodents generally develop a certain aversion to alcohol, and in the model, they have to receive large amounts of alcohol, so it is not the best way of projecting human alcoholic liver disease [43]. When comparing rodents, mice proved to be a better model than rats, especially strains HAP-2 and C57BL/6 [44,45]. The model inducing liver fibrosis with ethanol is one of the models that is often combined with an additional cause of fibrosis, which further enhances the effect of ethanol. Certain studies included, in addition to ethanol, the chemical factor of liver fibrosis CCL4, and it was determined that the combination of these two factors proved to be a more efficient model in the development of cirrhosis [46,47].

Today, a model that uses the consumption of high-fat food, with or without the presence of ethanol, is increasingly being used as a potential model for the development of liver fibrosis because it represents a simulation of the Western model of life. This model leads to an increase in the liver expression of TLR4, resulting in a strong immune response. Therefore, with this model, in addition to the mechanisms, inflammation can also be monitored [48]. The application of the diet-induced liver fibrosis model is limited due to the fact that the result does not reflect the true characteristics of the pathology in humans. Dietary protocols for the development of liver fibrosis are also applied to rats and mice, but the best and most sensitive model turned out to be the mouse C57BL/6 [49]. Additionally, similar to the use of ethanol, the use of fat-enriched food for the development of fibrosis models in combination with the chemical factor CCL_4_ results in a more efficient research model. With this combination, significant characteristics of non-alcoholic steatohepatitis develop, accompanied by steatosis, inflammation, and fibrosis.

In today’s liver fibrosis research, transgenic models are ubiquitous, especially in the study of signaling and metabolic pathways, but also in models of the development of viral infections as the cause of fibrosis [27,50]. Transgenic animals, usually mouse strains, are used, depending on the key genes to be investigated. Thus, MDR2^−^/^−^ mice represent a model of chronic cholestatic liver injury [51,52]. TGF-β1 transgenic mice enable the control of TGF-β1 protein expression, which participates in the control of cell growth, cell proliferation and differentiation, and apoptosis [53,54]. Protein 2-deficient mice develop a model that induces hepatocyte necrosis, inflammation, proliferation, and destruction of hepatic ducts by increasing the expression of TGFβ and markers of hepatic stellate cell activation [55]. Furthermore, Alms1 Fat Aussie mutant mice represent obese mice that possess an 11-base pair deletion in the Alms1 gene. In this type of mice, a high-fat diet promotes lipid formation in the liver, hepatocellular injury, and inflammation [56]. Infection can also develop with different parasites such as *Schistosoma mansoni* and *Schistosoma japonicum*, which cause the activation of hepatic stellate cells [57,58]. Such models of liver fibrosis are good models for examining the role of interleukins and cytokines in inflammatory processes.

Finally, the last form of liver fibrosis model that is very often used is surgical ligation of the bile ducts. It was initially applied to rats, but the model was also modified for mouse strains [59,60]. Surgical methods of producing liver fibrosis are suitable for short-term studies because bile duct ligation causes increased animal mortality after several weeks [61,62]. This model creates cholestatic liver injuries that result in fibrosis and an increase in the value of accompanying markers, inflammation of liver cells, accumulation of inflammatory cells in the portal tract, and an increase in oxidative stress [63,64,65]. All the advantages and disadvantages of the in vivo methods are summarized in Table 2.

There are also some animal models that have the ability to prevent fibrosis and enable tissue regeneration. For example, mice models Acomys cahirinus (spiny mouse) or Orbq13, due to their gene system that modulates susceptibility to causing fibrosis, do not cause the desired consequences, leading to tissue regeneration and prevention of the immune response [66,67]. Such mouse strains represent quality models for genomic analysis research that can help in assessing the development of fibrosis in humans.

## 3. Pharmacotherapeutic Strategies and New Targets in Liver Fibrosis

Various pharmacotherapeutic strategies for the treatment of liver fibrosis, along with promising new targets, are presented in the next few chapters, and summarized in Table 3.

### 3.1. Treatment of the Underlying Condition

The primary goal in the treatment of chronic liver disease is to eliminate any of the causative agents or conditions involved in constant parenchymal damage. Nowadays, chronic liver disease is mainly induced by hepatotropic viruses (hepatitis B virus (HBV) or hepatitis C virus (HCV)), excessive alcohol consumption (alcoholic liver disease (ALD)), or a metabolic disorder called NAFLD [61].

HCV infection is still one of the leading causes of chronic hepatitis and cirrhosis, affecting about 70 million people around the world [62]. Most infected individuals develop chronic infection, and 20–30% will develop liver cirrhosis within 30 years [63]. Fortunately, today, we have direct-acting agents (DAA), an efficient and safe therapeutic option characterized by >90% sustained viral response (SVR) [64]. Despite the virus eradication, patients with advanced fibrosis have a significant risk for developing hepatocellular carcinoma. Furthermore, recent studies demonstrate the persistence of epigenetic changes associated with an increased risk of HCC despite SVR, indicating that the viral cure does not eliminate the risk of fibrosis and cancerogenesis entirely [65,66].

Hepatitis B virus infection, affecting more than 250 million people worldwide, is characterized by a chronic course of disease, especially if the transmission is vertical [67]. Current therapeutic options are immunomodulatory agents (interferons) and antiviral agents (nucleoside and nucleotide analogues—NA) which enable a functional cure, loss of HBsAg, and/or HBV-DNA suppression, while viral DNA persists, incorporated in the host genome or as a form of stable episomal minichromosomes–circular DNA [68]. Thus, the eradication of the HBV virus remains a challenge due to the unique viral life cycle of HBV, making it a constant threat for disease reactivation and the development of liver cirrhosis and HCC.

The growing disease NAFLD still is a condition without any approved pharmacological treatment. Nowadays, only lifestyle modification (dietary intervention and physical activity) and bariatric surgery are proven interventions with any impact on disease course, while licensed therapeutic agents are lacking [69].

Taken together, the elimination of an inflammation inducer definitely is not sufficient for the reversion of liver fibrosis, and the development of complications implicate the need for the introduction of antifibrotic strategies.

### 3.2. Anti-Inflammatory Action and Liver Protection

Liver injury leads to an inflammatory response by activating inflammatory cells and releasing inflammatory mediators such as cytokines and chemokines that attract monocytes, macrophages, and lymphocytes to the injured area. Macrophages and Kupffer cells release apoptosis signal-regulating kinase 1 (ASK1), TNF-alpha, chemokines such as CC -chemokine ligand 2 (CCL2) and CC -chemokine ligand 5 (CCL5), and pan-caspase, which further enhance liver injury. Pan-caspase can lead to hepatocyte apoptosis through apoptotic protease activation, while ASK1 is involved in inflammation and apoptosis through the activation of the MAPK signal pathway [81,82]. De novo lipogenesis (DNL) is characterized by the excessive accumulation of fatty acids in the liver, which can lead to lipotoxicity and cause oxidative stress, inflammation, apoptosis of hepatocytes, liver injury, and subsequently, liver fibrosis [83]. Therefore, potential antifibrotic drugs currently in the clinical research phase target inflammatory pathways and lipotoxicity via small-molecule agonists, antagonists, antibodies, and proteins.

Cenicriviroc (CVC) is a chemokine receptor CCR2/5 antagonist with antifibrotic activity in adults with NASH. A phase 2b CENTAUR study has shown improvement in liver fibrosis with no effect on steatohepatitis, suggesting a combination of CVC with other molecule compounds that affect the metabolism of NASH [69]. In a recent study, Puengel et al. demonstrated a potential therapeutic strategy by combining two pharmacological agents in a mouse model of NASH [84]. They used a CCR2/CCR5 antagonist as an antifibrotic agent and an FGF21 (fibroblast growth factor) analog as a metabolic agent to compare the effect of single-drug therapy with combined therapy, and showed that combined therapy was superior to single-drug therapy in improving liver inflammation and fibrosis [84]. Future studies in the human NASH model are needed.

In a multicenter randomized phase 2 clinical trial, Loomba et al. evaluated the safety and efficacy of selonsertib, a selective inhibitor of ASK1, alone or in combination with simtuzumab in patients with NASH and stage 2 or 3 liver fibrosis. The results showed the regression of fibrosis in a high proportion of patients, suggesting that selonsertib is a potential antifibrotic drug [70]. On the contrary, two randomized, double-blind, placebo-controlled, phase 3 clinical trials of selonsertib in NASH patients and F3 or F4 failed to reach ≥1-stage fibrosis improvement in a substantial proportion of patients. Although there were no significant differences between the selonsertib and placebo groups, the study revealed a significant reduction in p38 phosphorylation, suggesting that the pharmacodynamic activity of selonsertib is achieved through the inhibition of ASK1 [85].

Emricasan is a pan-caspase inhibitor that can contribute to the reduction in hepatic inflammation and hepatocyte apoptosis by lowering serum levels of aminotransferases and inflammatory markers in NAFLD patients [71]. A randomized, double-blind, placebo-controlled trial of 217 patients with decompensated NASH cirrhosis treated with 5 mg or 25 mg emricasan or placebo showed that emricasan was safe and well tolerated but clinically ineffective [71]. However, there was a statistically significant decrease in caspase-related biomarker activity in the 25 mg-dose group compared with the 5 mg-dose and placebo group, suggesting that the drug dosage may not have been appropriate for these patients [86]. Weinberg et al., in their multicenter, randomized, double-blind control trial, tried to evaluate the effect of emricasan on post-SVR liver fibrosis in HCV patients after liver transplantation [87]. The result showed that emricasan is well tolerated and safe in combination with immunosuppressants and that patients with moderate liver fibrosis after HCV eradication may benefit from the therapy [87].

### 3.3. Interference or Blockage of HSC Activation and Proliferation

Hepatic fibrosis is initiated and progressed by the complex process of activated HSCs differentiating into myofibroblasts and producing excessive extracellular matrix proteins [88]. The activation and proliferation of HSCs is regulated by several signaling pathways, some of which can be targeted to inhibit fibrogenesis [89]. The TGF-β/Smad signaling pathway is an intracellular signaling cascade that plays a critical role in the activation of HSCs and the subsequent development of liver fibrosis [90]. The activation of the TGF-β/Smad signaling pathway by sphingosine-1-phosphate can induce fibrosis in the liver [91]. Miglustat is a drug that inhibits the synthesis of glycosphingolipids and has been successfully used in Gaucher disease type 1 and Niemann–Pick disease type C [92,93]. A recent study shows that miglustat reverses and prevents fibrosis by inhibiting TGF-β/Smad signaling in HSCs and CCl4-treated mice [72]. The established link between type 2 diabetes mellitus (T2DM) and NAFLD has led to numerous studies suggesting that anti-diabetic drugs could offer benefits in treating NAFLD [94,95]. Evogliptin is a medication that is classified as a selective dipeptidyl peptidase-4 inhibitor, significantly reduces the expression of α-smooth muscle actin (α-SMA) and collagen I, and inhibits HSC activation [73,74]. Empagliflozin (EMPA), a sodium–glucose cotransporter inhibitor, significantly reduces the activation of HSCs and the expression of profibrogenic genes such as α-SMA, TGF-β cytokine, and collagen 1 α1, and prevents fibrosis by a decrease in the total of Yes-associated protein (YAP) level and its activation by the phosphorylation. Moreover, EMPA suppresses the proliferation of activated HSCs in vivo and in vitro through the activation of the Hippo/YAP signaling pathway [75]. The dysregulation of the Hippo/YAP signaling pathway is associated with liver fibrosis and hepatocellular carcinoma (HCC) [96]. Kim et al. reported that HL3501, a new selective adenosine A3 receptor antagonist, is capable of reducing the expression of profibrotic markers including α-SMA, collagen I, and fibronectin in activated HSCs [76]. This suggests that targeting adenosine receptors may represent a potential therapeutic strategy for preventing fibrosis in liver diseases. These findings build upon the previous research by Perez-Aso et al., which demonstrated the role of the Adenosine 2A receptor in promoting collagen production in human fibroblasts [97]. Aspirin is a non-steroidal anti-inflammatory drug used to alleviate pain and inflammation, and it has been reported to prevent fibrosis [98]. Sun et al. discovered that aspirin reduced the protein levels of α-SMA, collagen I, TGF-β1, p-Smad2, and p-Smad3 in vitro, which was supported by in vivo experiments [77].

Therefore, targeting HSC activation and proliferation is a promising strategy for the treatment of liver fibrosis. Developing drugs that selectively inhibit HSC activation and proliferation without affecting their other physiological functions could be a potential therapeutic approach for liver fibrosis.

### 3.4. Inhibition of the ECM Production and Promotion of ECM Degradation

ECM proteins play a crucial role in the development of liver fibrosis as they cause a distortion in the liver architecture [99]. Under normal conditions, there is a balance between the degradation and production of ECM which leads to HSCs being responsible for the production of ECM. In liver fibrosis, HSCs are the main cause of the uncontrolled production and degradation of ECM, which eventually results in liver injury [82,100]. Moreover, when it comes to the regulation of ECM degradation, matrix metalloproteinases (MMPs) are enzymes responsible for the proper degradation of ECM components. In liver injury, these enzymes are inhibited by tissue inhibitors of metalloproteinase (TIMPs) [10,100,101]. In a significant manner, the altered remodeling of ECM is affected by an increase in the expression of TIMPs which prevent the degradation of ECM via MMP inhibition [78]. A novel therapy used curcumin-/chitosan-coated green silver nanoparticles to induce the inhibition of TIMPs, as it is known that curcumin regulates various inflammatory signaling pathways. The results indicated that curcumin plays an important role in the downregulation of TIMPs as it decreases the expression of TIMP1 genes in vivo. On the other hand, the transforming growth factor-β (TGF-β) family serves as a regulator of both cell differentiation and cell proliferation, as well as ECM production [102]. Another important factor involved in the regulation of ECM synthesis is the connective tissue growth factor (CTGF). CTGF is a protein mainly generated by activated HSCs, even though hepatocytes and portal fibroblasts also induce its production. This growth factor acts as a downstream effector of TGF-β, and the inhibition of CTGF is known to abolish the excessive ECM production mediated by TGF-β [103,104]. A therapy using Bradykinin 1 receptor antagonist (BI 113823), the main inhibitor of Kinin B1 receptors (B1Rs), has been shown to reduce the expression of both CTGF and TGF-β in vivo. Additionally, the results of this study confirmed that BI 113823 inhibits the activation of HSC as its activation is originally stimulated by both TGF-β and B1R agonists [79]. Therefore, as HSCs are the main CTGF-producing cells, they show great potential as promising targets for the future treatment of liver fibrosis. Additionally, the induction of ECM degradation could play a vital role in further treatments via the activation of MMPs and/or inhibition of TIMPs.

### 3.5. Gene Therapy

ECM is the main treatment target for the reversal of liver injury in patients suffering from severe liver fibrosis. Few novel therapies for liver fibrosis are directed toward the regulation of ECM production and degradation via gene therapy [105]. RNA interference (RNAi) is a gene-silencing therapy characterized by small interfering RNAs (siRNAs) which inhibit the targeted gene [106]. Currently, there are several siRNA modifications which include siRNA conjugated with N-acetylgalactosamine (GalNac) and lipid-based nanoparticles (LNPs) which make the delivery of siRNA less difficult [25,107,108]. The LNP-based siRNA, specifically, shows great potential for gene silencing in liver fibrosis. Moreover, one such therapy was used in order to inhibit the heat shock protein 47 (HSP47) produced by HSC. HSP47 was silenced in vivo via anisamide-tethered lipidoid AA-T3A-C12/siHSP47 LNP, which led to ~65% silencing of HSP47 and a reduction in fibrotic alterations [25,26]. Furthermore, adeno-associated virus (AAV)-mediated gene therapies have also shown great potential as a therapy for liver fibrosis. Few AAV-mediated gene therapies were conducted using transgenes hepatocyte growth factor (HGF), bone morphogenetic protein 7 (BMP7), and microRNA-19b (miRNA-19b), where all of them were confirmed to assess the resolution of liver fibrosis [80]. HGF was proven to inhibit the expression of profibrotic genes and to upregulate the expression of MMP13, whereas BMP7 and miR-19b improved liver function by inhibiting the activation of HSC [80,109]. Therefore, both siRNA and AAV-mediated gene therapies have the potential to be used as treatment strategies in other liver diseases, as gene therapies are proven to be effective in the reversal of liver fibrosis.

The advantages and disadvantages of numerous abovementioned treatments are summarized in Table 4.

## 4. Conclusions

Taken all together, although it is one of the greatest challenges in the current medical research and practice, fibrosis is accordingly being approached by numerous researchers. As a result, various in vitro and in vivo fibrosis models have been proposed and established, followed by numerous pharmacotherapeutic solutions. These steps represent a great shift to a better diagnosis and treatment of this disease, the prevention of its progression to irreversible cirrhosis and HCC, and the enhancement of life quality and duration in a great number of patients worldwide. Nevertheless, more research is necessary in order to introduce these diagnostic and pharmacotherapeutic tools into everyday clinical practice.

## Figures and Tables

**Table 1:** Cell types used for the establishment of the liver fibrosis model, and their main characteristics.

Cell Type	Characteristics	References
Murine cell line (GRX); primary rodent hepatic stellate cells	First-described in vitro liver fibrosis model. Originating from cirrhotic fat-storing cells and normal fat-storing cells.	[24]
Human hepatic stellate cells (LI90)	First human HSC line. Derived from an epithelioid hemangioendothelioma; good model for the observation of drug targets in HSC activation; deterioration process after a few passages.	[24]
Primary hepatic stellate cells	Originating from normal liver tissue; used as a model for the screening of pro- and antifibrosis compounds; represent a significant reflection of in vivo liver fibrosis; their life span is limited; responsible for vitamin A storage to a profibrogenic myofibroblast phenotype.	[24]
Liver sinusoidal endothelial cells, Kupffer cells, and stellate cells	Non-parenchymal cells; preferred in in vitro toxicity studies and drug-induced liver fibrosis in humans.	[24]
Primary human hepatocytes	Rapidly lose their metabolic capacity when placed in 2D; preferred in in vitro toxicity studies and drug-induced liver fibrosis in humans.	[24,25,26]

**Table 2:** Advantages and disadvantages of in vivo methods.

Method	Advantages	Disadvantages
Carbon Tetrachloride (CCL4)	- the most commonly used hepatotoxin in the study of liver fibrosis and cirrhosis - it can be applied to rats and mice (preference is shown to mice due to their higher metabolic rate of CCl4 compared to rats) - useful model for studying the mechanism of spontaneous reversal of liver fibrosis - relatively cheap method	- oral administration causes high mortality
Thioacetamide (TAA)	- application in rat and mouse models (a more suitable method for rats) - widely used as a model for inducing experimental liver fibrosis - used to evaluate the therapeutic effect of liver fibrosis	- CYP2A5 may have a protective effect against TAA-induced liver injury and fibrosis
Dimethylnitrosamine and diethylnitrosamine	- due to the stability of liver changes, a frequently used experimental model - represents a model similar to early human cirrhosis - a simple model for the development of fibrosis	- toxicity
Ethanol	- often combined with an additional cause of fibrosis	- often combined with an additional cause of fibrosis - rodents generally develop a certain aversion to alcohol - variability between animals
High-fat food	- developing obesity as an additional parameter for study - more effective in combination with other models for the development of liver cirrhosis	- more effective in combination with other models for the development of liver cirrhosis - result does not reflect the true characteristics of the pathology in humans - long-term process of developing symptoms
Transgenic models	- a useful model that can be used to study mechanisms and inflammation - the possibility of using a model with specific characteristics that we want to observe (gene inhibition, obesity, etc.)	- long-term process of developing symptoms
Surgical method	- very often used model - well applicable in the rat model (also modified for mice)	- suitable for short-term studies

**Table 3:** Overview of the therapeutic options.

The Main Mechanism of Action	Therapeutic Agent	References
Treatment of the underlying condition	Direct-acting agents	[24]
	Interferons and nucleoside and nucleotide analogues	[68]
Anti-inflammatory action and liver protection	Cenicriviroc	[69]
	Selonsertib	[70]
	Emricasan	[71]
Interference or blockage of HSC activation and proliferation	Miglustat	[72]
	Evogliptin	[73,74]
	Empagliflozin	[75]
	Selective adenosine A3 receptor antagonist (HL3501)	[76]
	Aspirin	[77]
Inhibition of ECM production and promotion of ECM degradation	Curcumin-/chitosan-coated green silver nanoparticles	[78]
	Bradykinin 1 receptor antagonist (BI 113823)	[79]
Gene therapy	N-acetylgalactosamine and lipid-based nanoparticles	[25,26]
	Adeno-associated virus-mediated gene therapy	[80]

HSCs—hepatic stellate cells, ECM—extracellular matrix.

**Table 4:** Advantages and disadvantages of numerous pharmacotherapeutic options.

Treatment	Advantages	Disadvantages
CVC	Improves F2-F3 liver fibrosis, prevents liver cirrhosis, reduces inflammation biomarkers, has good safety and resistance.	Phase 3 clinical trial showed a lack of efficacy, an uncertain role in patients with mild liver fibrosis [24].
Selonsertib	Improves F2-F3 liver fibrosis.	Phase 3 clinical trial in patients with F3 liver fibrosis showed lack of efficacy [24].
Emricasan	Reduces hepatic inflammation and hepatocyte apoptosis, safe and well tolerated in combination with immunosuppressants.	Clinically ineffective.
Miglustat	Prevents and reverses liver fibrosis in human hepatic stellate cells and carbon tetrachloride (CCl4)-treated mice through TGF-β/Smad pathway suppression in HSCs.	Not specific to hepatic stellate cells (HSCs) and may have effects on other cell types in the liver or in other organs. This could potentially lead to off-target effects or unwanted side effects.
Evogliptin	Direct inhibition of inflammatory and fibrotic signaling in isolated liver cells.	While preclinical studies have shown promising results, further studies are needed to evaluate the safety and efficacy of evogliptin for the treatment of liver disease in humans.
Empagliflozin	Reduces the progression of hepatic fibrosis in mouse models through inhibition of the Hippo signaling pathway.	While preclinical studies have shown promising results, further studies are needed to evaluate the safety and efficacy of empagliflozin for the treatment of liver disease in humans.
Selective adenosine A3 receptor antagonist (HL3501)	Inhibits the expression of profibrotic markers in human hepatic stellate cells.	Lack of clinical studies. Possible that HL3501 could have unintended or off-target effects when used in humans. Animal models may not fully reflect human disease.
Aspirin	Decreases the expression of profibrotic markers in rat model of liver fibrosis. Aspirin is a well-established medication with a known safety profile, especially when used in low doses.	Further studies are needed to determine the optimal dosage, duration, and safety profile of aspirin treatment for liver fibrosis in humans. Animal models may not fully reflect human disease.
Curcumin-/chitosan-coated green silver nanoparticles	Decrease expression of TIMP1 genes.	Low solubility and bioavailability of curcumin [24].
Bradykinin 1 receptor antagonist (BI 113823)	Decreases expression of CTGF, TGF-β, and B1R.	B2R expression is not affected [25,26].
Lipid-based nanoparticles	Improve delivery of siRNA.	This study used 3T3-GFP fibroblasts instead of liver-resident HSCs.
Adeno-associated virus mediated gene therapy	Inhibits activation of HSCs.	No reported clinical trials.

## Data Availability

The data presented in this study are available on request from the corresponding authors.
